# Moving toward a higher efficiency of microcell-mediated chromosome transfer

**DOI:** 10.1038/mtm.2016.43

**Published:** 2016-06-22

**Authors:** Mikhail Liskovykh, Nicholas CO Lee, Vladimir Larionov, Natalay Kouprina

**Affiliations:** 1 Developmental Therapeutics Branch, National Cancer Institute, Bethesda, Maryland, USA

## Abstract

Microcell-mediated chromosome transfer (MMCT) technology enables individual mammalian chromosomes, megabase-sized chromosome fragments, or mammalian artificial chromosomes that include human artificial chromosomes (HACs) and mouse artificial chromosomes (MACs) to be transferred from donor to recipient cells. In the past few decades, MMCT has been applied to various studies, including mapping the genes, analysis of chromosome status such as aneuploidy and epigenetics. Recently, MMCT was applied to transfer MACs/HACs carrying entire chromosomal copies of genes for genes function studies and has potential for regenerative medicine. However, a safe and efficient MMCT technique remains an important challenge. The original MMCT protocol includes treatment of donor cells by Colcemid to induce micronucleation, where each chromosome becomes surrounded with a nuclear membrane, followed by disarrangement of the actin cytoskeleton using Cytochalasin B to help induce microcells formation. In this study, we modified the protocol and demonstrated that replacing Colcemid and Cytochalasin B with TN-16 + Griseofulvin and Latrunculin B in combination with a Collage/Laminin surface coating increases the efficiency of HAC transfer to recipient cells by almost sixfold and is possibly less damaging to HAC than the standard MMCT method. We tested the improved MMCT protocol on four recipient cell lines, including human mesenchymal stem cells and mouse embryonic stem cells that could facilitate the cell engineering by HACs.

## Introduction

Microcell-mediated chromosome transfer (MMCT) technique was developed in 1977 by Fournier and Ruddle.^1^ It enables a single, intact mammalian chromosome or an autonomous megabase (Mb)-sized chromosome fragment to be transferred from donor to recipient cell lines. Typical donor cells used in MMCT are Chinese hamster ovary (CHO) and mouse A9 cells. Unlike most cell lines, which die under prolong exposure to microtubule inhibitors, the A9 and CHO cells undergo repetitive hyperploidization in the presence of Colcemid with micronucleation occurring during the transition from metaphase to pseudo-G1.^2^ Micronuclei formed by A9 and CHO are thus smaller and more numerous. These micronuclei can subsequently be extruded from the cell as microcells by centrifugation in the presence of cytoskeleton disruptor, like an actin inhibitor.^3^ Several groups constructed mouse A9 or CHO-microcell hybrid libraries containing individual human chromosomes that provided valuable resources for mapping and functional studies of human genes.^4–7^

Alternatives to MMCT include a method described by Mullinger’s group^8^ in 1975. Like MMCT, this method also aimed to transfer chromosomes between cell lines. However, it differs by the manner it generates chromosome containing membrane bound particles. Unlike MMCT, the method described by Mullinger and colleagues induces mitotic cells to generate mini-segregants, cluster of small daughter cells. It induces these abnormal mitotic chromosome segregations by storing mitotic cells at 4 °C, followed by resumption of growth upon return to 37 °C incubation.

Although the MMCT method was developed nearly 40 years ago, two main limitations make the method tedious. First, the frequency of the chromosome transfer from donor cells into recipient cells is very low. Second, MMCT is not universally applicable, particularly in cell lines where fusion with microcells is very inefficient. Yet despite these limitations, MMCT technique has been applied to various studies over the years. For example, MMCT has contributed to mapping the genes through functional complementation, *i.e.*, genes for tumor suppression,^9^ DNA repair,^10–12^ metastasis, telomerase regulation, genomic instability,^13^ mitochondrial disorders,^14^ and lysosomal storage diseases.^15^ MMCT technique has been also applied for analysis of specific chromosome status such as aneuploidy and epigenetics.^4,16,17^ A relatively new MMCT application is the transfer of mammalian artificial chromosomes (that include mouse artificial chromosome (MAC) and human artificial chromosome (HAC) vectors) into recipient cells for gene function studies and other goals.^18–25^ The advantage of artificial chromosomes over entire chromosomes transfer is that MAC and HAC vectors can carry an individual gene of interest, while natural chromosomes contain numerous genes, which would complicate the study a single specific gene.

Mammalian artificial chromosomes have been generated by either a bottom-up approach (*de novo* creation) or a top-down approach (truncation of natural chromosomes).^18–25^ MACs and HACs are maintained stably as additional chromosomes in mammalian cells over multiple generations due to the presence of a functional kinetochore. Some modified MACs and HACs contain a single gene-loading site that allows insertion a therapeutic gene up to several Mb in size.^26–28^ Recently, MACs/HACs were constructed with multiple gene-loading sites^29–31^ to carry several genes or assemble a single large gene along with its native gene regulatory elements. Many publications have described the application of MMCT to transfer MACs/HACs carrying certain genes to gene-deficient cells for gene function studies, humanized animal transgenesis, and high-yield protein production.^21–25,28,32–37^ As MMCT has numerous applications, a more efficient procedure for chromosomes or MACs/HACs transfer is a worthy goal.

In this study, we optimized the MMCT protocol and demonstrated that replacement of key reagents, *i.e.*, Colcemid, a microtubule inhibitor that arrests cells at metaphase, and Cytochalasin B, an actin inhibitor that induces actin cytoskeleton disassembling, and a Collagen/Laminin coating to improve cell adherence to the culture flask’s surface significantly improved the accuracy and efficiency of MMCT.

## Results

### Experimental design

**Figure 1:**
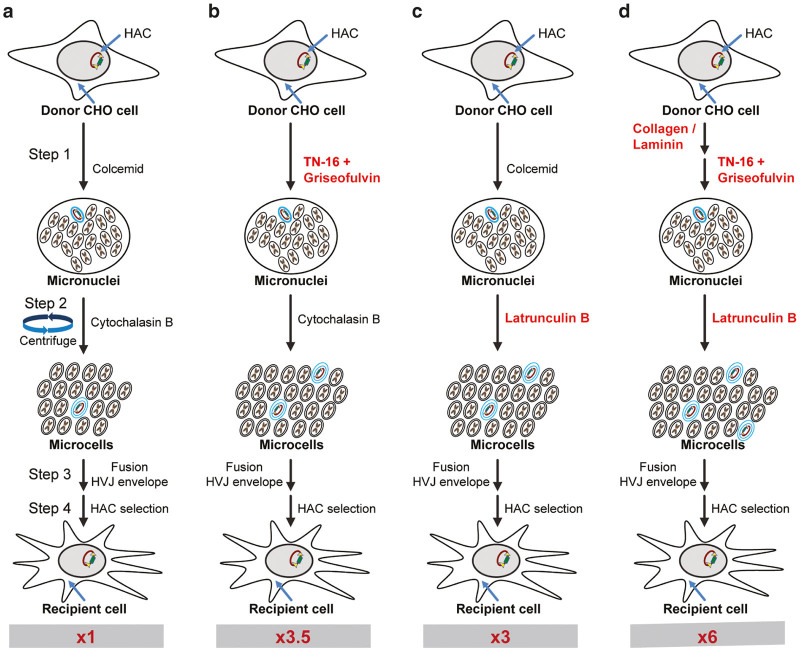
Diagram of the original and modified microcell-mediated chromosome transfer (MMCT) techniques. (**a**) The original protocol using Colcemid and Cytochalasin B as key chemicals. The cells are cultured on standard treated tissue culture plastic. (**b**) The protocol is identical to the original aside from Colcemid being replaced by TN-16 + Griseofulvin combination. (**c**) The protocol is the same as the original protocol aside from Latrunculin B replacing Cytochalasin B. (**d**) A modified MMCT protocol that includes replacement of both key chemicals by TN-16 + Griseofulvin and Latrunculin B. The cells are culture on plastic coated with Collagen/Laminin. Red numbers at the bottom of each panel indicates the relative increase in efficiency compared with the original procedure. Steps 1, 2, and 3 correspond to steps in the modified protocol of MMCT described in detail in Supplementary Materials.

The original MMCT protocol^1^ has barely changed in decades, and it includes three major steps. Step 1 is the treatment of donor cells with a cytostatic chemical (Colcemid) to stop the cells at the metaphase phase stage of cell cycle for 48–96 hours (Figure 1a). During this prolong incubation at metaphase, the cells become micronucleated, where each chromosome becomes surrounded with a nuclear membrane. In step 2, adherent micronucleated cells are centrifuged in the presence of a chemical-inducing disarrangement of the actin cytoskeleton (Cytochalasin B) (Figure 1a). The breakdown of the actin cytoskeleton makes the cell more fluid, which aids in the extrusion of micronuclei from the cell by the force imposed via centrifugation to form microcells. Each microcell is a chromosome surrounded by both a nuclear membrane and cytoplasmic membrane. In step 3, the microcells are fused to the recipient cells using agents such as polyethylene glycol or hemagglutinating virus of Japan viral envelope (Cosmo Bio) (Figure 1a, Supplementary Figure S1). Based on the logic of the protocol, the efficiency of MMCT should correlate with the number of metaphases (step 1) formed during metaphase block and the degree of actin cytoskeleton disassembly during micronuclei extrusion that forms microcells (step 2).

**Figure 2:**
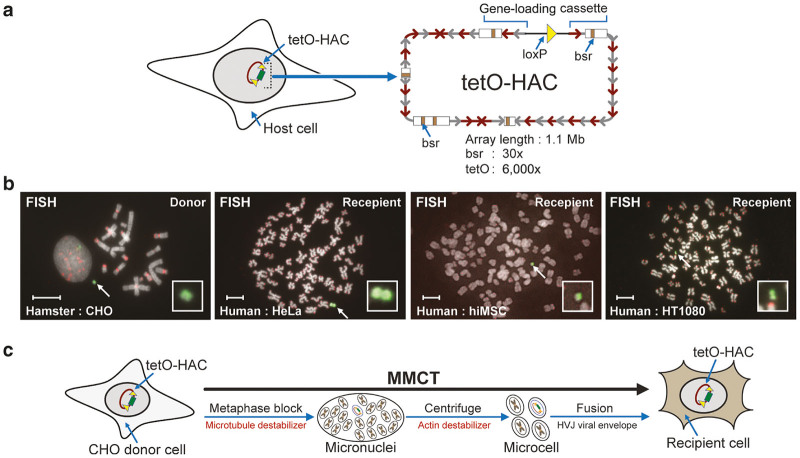
Human artificial chromosome (HAC). (**a**) Diagram of the alphoid^tetO^-HAC^37^ (^tetO^-HAC) used in this study. The HAC contains a unique gene-loading loxP site.^40^ The HAC consists of ~6,000 copies of the 42-bp tetracycline operator (tetO) sequence incorporated into every second alphoid DNA monomer of the 1.1 megabase-size alphoid DNA array.^37,43^ The HAC has ~30 copies of the selectable marker blasticidin (bsr). (**b**) FISH of a donor hamster CHO cells and recipient human cell lines carrying the autonomously propagated HAC. FISH analysis was performed using the PNA-labeled probe for tetO-alphoid sequences (in green) and telomeres (in red) (see details in Materials and Methods). The HAC is indicated by arrow. The scale bar is 10 µm. (**c**) The microcell-mediated chromosome transfer (MMCT) transfer of alphoid^tetO^-HAC from donor hamster CHO cells to human recipient cells. The individual steps of MMCT transfer include metaphase block, micronuclei and microcell formation, and fusion of microcells with recipient cells using the hemagglutinating virus of Japan (HVJ) viral envelope.

In this study, we used the alphoid^tetO^-HAC, a synthetic chromosome 1.1 Mb in size, that contains a blasticidin selectable marker (bsr) (Figure 2a).^24,38,39^ This HAC is on the frontier of human kinetochore studies and has been used for gene function analyses and creation of transchromic animals.^32,33,40^ In hamster, mouse, and human cells, this HAC is stably maintained as an autonomous chromosome (Figure 2b).^33,34,38,39,41–43^ We transferred the alphoid^tetO^-HAC from donor hamster CHO cells to different recipient cell types (Figure 2c) to gauge if a modification to the MMCT protocol was an improvement. In our experiments, we compared the material derived from six flasks.

### Replacement of Colcemid with TN-16 + Griseofulvin resulted in approximately fourfold increase in MMCT efficiency

Colcemid is a well-known microtubule inhibitor. It binds to plus ends of microtubules and suppresses microtubule dynamics. This suppression prevents formation of the mitotic spindle, allowing cells to be captured at metaphase.^44^

From our observations, hamster CHO cells are very resilient to Colcemid treatment (Supplementary Table S1). Only 3–4% of treated CHO cells are arrested at metaphase with Colcemid treatment. This is severe limitation of the substrate available for micronuclei formation. To overcome this problem, we attempted to find a better metaphase blocker. We treated CHO cells with different microtubules inhibitors, some of which targeted the same site as Colcemid, while others targeted different domains within the microtubule dimer at various concentrations and combinations (Supplementary Tables S1–4). The best combination was 50 µmol/l Griseofulvin + 160 µmol/l TN-16 (Supplementary Table S2). It increased metaphase formation to an average of 15%, approximately four times higher compared with the standard 100 ng/ml of Colcemide (χ^2^(1) = 91.02, *P* < 0.0001).

**Figure 3:**
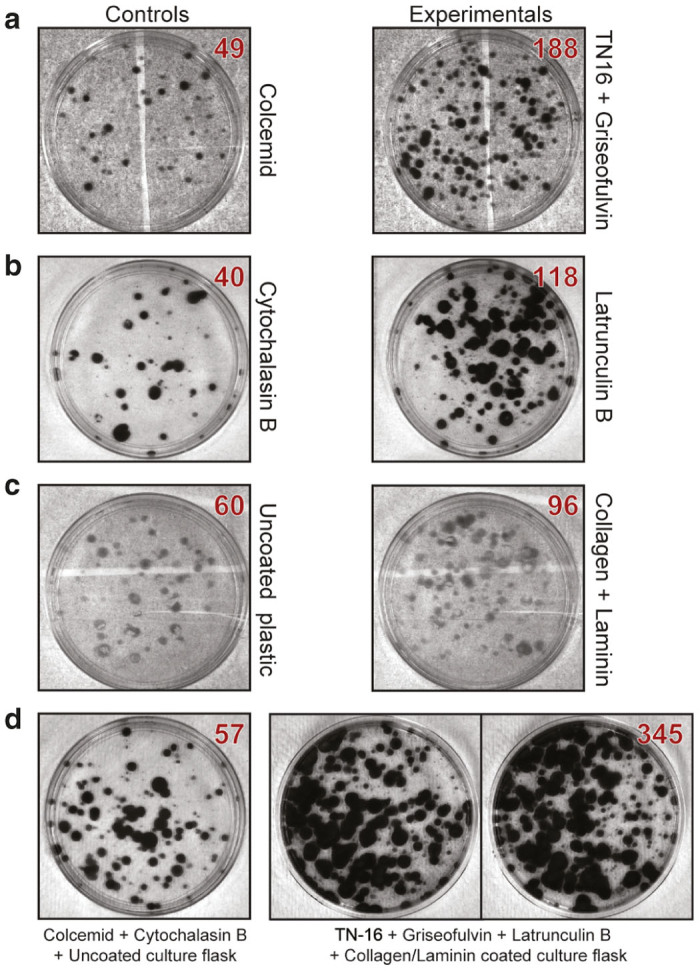
Colonies of human fibrocarcoma HT1080 cells containing the human artificial chromosome (HAC) after microcell-mediated chromosome transfer (MMCT) under different conditions. (**a**) Comparison of MMCT efficiency using Colcemid (left panel) and TN-16 + Griseofulvin combination (right panel). (**b**) Comparison of MMCT efficiency using Cytochalasin B (left panel) and Latrunculin B (right panel). (**c**) Comparison of MMCT efficiency using unmodified cell culture flasks (left panel) and Collagen/Laminin covered flasks (right panel). (**d**) Comparison of MMCT efficiency using Colcemid + Cytochalasin B + unmodified cell culture flasks (original protocol) versus TN-16 + Griseofulvin + Latrunculin B + Collagen/Laminin covered flasks (new protocol). Red numbers at right corners of every picture indicate the number of colonies on the flasks. The controls in Figure 3a–d used the same condition as the standard MMCT procedure. The experiment in **a** replaced Colcemid with TN-16 and Griseofluvin with all other conditions remaining the same as in the standard MMCT procedure. The experiment in **b** replaced Cytochalasin B with Latranculin B with all other conditions remaining the same as in the standard MMCT procedure. The experiment in **c** uncoated plastic was replaced by plastic coated with Collagen + Laminin with all other conditions remaining the same as in the standard MMCT procedure. The experiment in **d** is the combination of all modifications.

**Figure 4:**
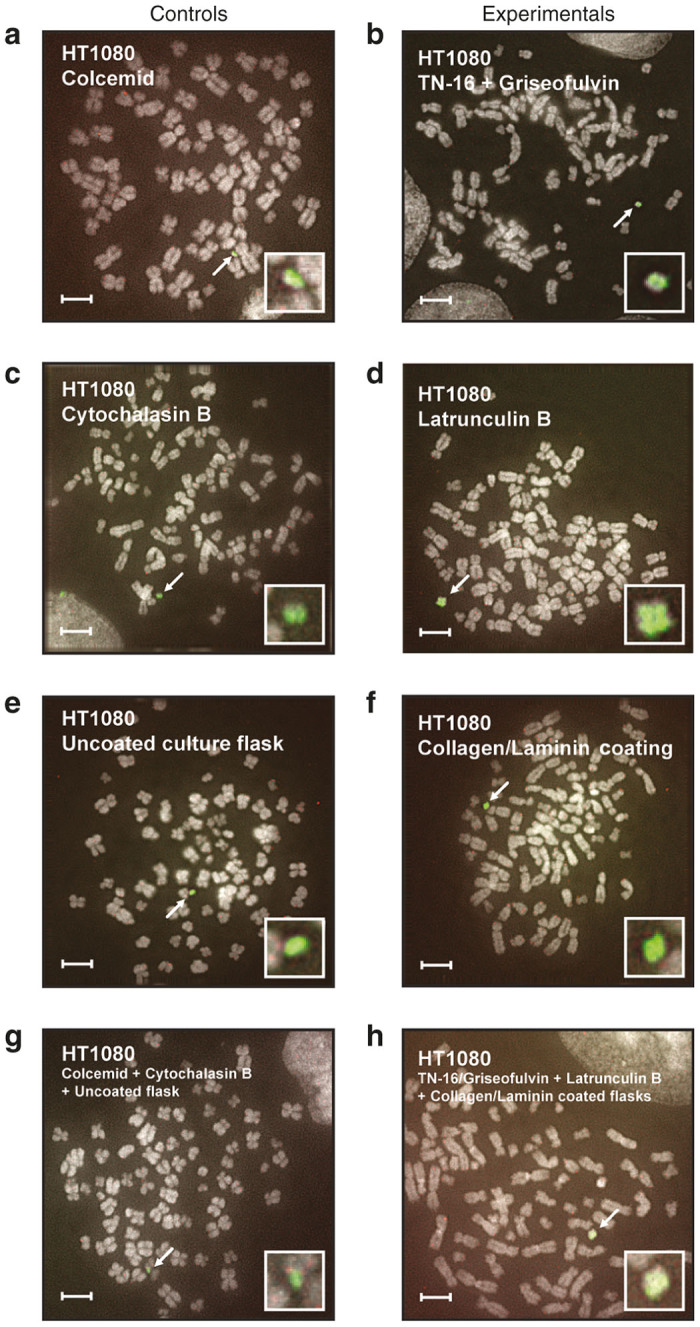
FISH analysis of human fibrosarcoma HT1080 cells after microcell-mediated chromosome transfer (MMCT) human artificial chromosome (HAC) transfer under different conditions. Examples of FISH metasphase obtained from experiment that compared the standard MMCT as a control (**a**, **c**, **e**, **g**) with modified conditions (**b**, **d**, **f**, **h**). The variable studied within each experiment is highlighted. FISH metaphase obtained from MMCT experiments between (**a**) Colcemid versus (**b**) TN-16 + Griseofulvin. FISH metaphase obtained from (**c**) Cytochalasin B versus (**d**) Latrunculin B experiments. FISH metaphase obtained from (**e**) uncoated culture flask versus (**f**) Collagen/Laminin coating experiments. FISH metaphase obtained from (**g**) the standard MMCT protocol (Colcemid + Cytochalasin B + Uncoated culture flasks) versus (**h**) the new MMCT protocol (TN-16/Griseofulvin + Latrunculin B + Collagen/Laminin coated flasks). Chromosomal DNA was counterstained with 4′,6-diamidino-2-phenylindole (white). The HAC was labeled green by FITC–PNA probe (see Materials and Methods for details) and highlighted within a white rectangle. The scale bar is 10 µm.

We changed step 1 in the protocol and incubated donor cells with combination of TN-16 + Griseofulvin instead of Colcemid (Figure 1b). After that, we proceeded with the standard MMCT protocol. We compared the number of colonies appeared after Colcemid treatment versus TN-16 + Griseofulvin treatment (Figure 3a) and verified that the HAC retains its autonomous form by Fluorescence in situ hybridization (FISH) (Figures 4a,b). We showed that replacing Colcemid with TN-16 + Griseofulvin combination significantly increased the efficiency of MMCT in three different human cell lines by ~3.7 times (Table 1) (ANOVA *F*(1,6) = 80.35, *P* = 0.0001).

### Replacement of Cytochalasin B with Latrunculin B increases MMCT efficiency threefold

The second key drug component of the original MMCT protocol is Cytochalasin B, which disassembles the actin cytoskeleton and allows extrusion of micronuclei to form microcells. Cytochalasin B is an old drug, and it inhibits actin filament polymerization through binding to the fast-growing (barbed) end of F actin filaments.^45,46^

We selected five compounds, Cytochalasin D, Chaetoglobosin A, Latrunculin B, Swinholide A, and Wiskostatin, that according to the literature targets various parts of actin filament and rated them against Cytochalasin B using an assay to test for actin cytoskeleton disarrangement inhibitors.^47^ From actin inhibition test, we identified three potential actin cytoskeleton disarrangement inhibitors that were better or equal to Cytochalasin B—Cytochalasin D, Chaetoglobosin A, and Latrunculin B (Supplementary Figure S2, green frames). Surprisingly, Cytochalasin D and Chaetoglobosin A works at concentrations 10 times less than Cytochalasin B, while Latrunculin B works at concentrations 100 times less than Cytochalasin B.

We then compared MMCT efficiency between Cytochalasin B and these three inhibitors. We changed step 2 in the standard MMCT protocol and incubated donor cells with one of candidate inhibitors using Cytochalasin B as a control (Figure 1c). We counted the number of blasticidin-resistant colonies containing the alphoid^tetO^-HAC that was obtained (Figure 3b). The presence of the HAC in recipient cells was confirmed by FISH analysis (Figures 4c,d). Latrunculin B showed the strongest effect. Replacement of Cytochalasin B by Latrunculin B significantly increased the efficiency of MMCT by threefold (Table 2) (*F*(2, 6) = 12.83, *P* < 0.0001).

As Latrunculin B works at a concentration of 0.2 µmol/l, 100 times lower than Cytochalasin B (20 µmol/l), it represents a significant reduction in the cost of MMCT experiments. As an example, in 2016, Santa Cruz Biotechnology sold 5 mg of Cytochalasin B (Cat: sc-3519; molecular weight = 479.61) at $104 and 1 mg of Latrunculin B (Cat: sc-203318; molecular weight = 395.51) at $216. Thus, the working solutions of 20 µmol/l Cytochalasin B cost $199.52/l, while 0.2 µmol/l Latrunculin B cost $17.09/liter. We have reused 0.2 µmol/l Latrunculin B solution twice and 20 µmol/l Cytochalasin B solution eight times with no loss of MMCT efficiency. It is also worth noting that the Cytochalasin B solution has been reported to be reusable up to 30 times. However, we filter our working solutions to prevent microcell cross contamination between experiment, so the cost of the filter system should be considered. A case of twelve 500 ml Corning Disposable Sterile Filter Systems (Cat: 430770; Fisher Scientific) cost $200.21 or $16.68 per filter. Hence, it is cost competitive to use new solutions of 0.2 µmol/l Latrunculin B for every new experiment.

### Replacement of the plastic surface by Collagen/Laminin coating resulted in a 1.6-fold increase in MMCT efficiency

It has long been observed that cells in metaphase round up and are easily detached from the plate surface. There is even a method to isolate metaphase cells by shaking culture plates. As formation of microcells is believed to be caused by extrusion of micronuclei from donor cells by centrifugation, we guessed that adhering more metaphase cells to the culture flask surface would improve the microcells production. Therefore, we compared the percentage of metaphase cells obtained from surfaces coated by four different cell adhesion proteins (Fibronectin, Laminin, Polylysine, and Collagen) singularly and in combinations against the tissue culture plastics that is normally used.

A small but statistically significant (Fisher exact test: *P* = 0.0020; two tailed) increase (1.3-fold) of attached metaphase cells was detected from plates coated by Collagen and Laminin (Supplementary Table S5). A corresponding improvement in MMCT efficiency (1.6-fold) also was observed (Figure 3c, Supplementary Table S6). The presence of an autonomous HAC in recipient cells was confirmed by FISH (Figures 4e,f).

### The optimized MMCT protocol: putting together TN-16 + Griseofulvin + Latrunculin B + Collagen/Laminin coating

After successful change of the original protocol’s key components, we decided to combine MMCT modifications from step 1 and step 2. When TN-16 + Griseofulvin + Latrunculin B were used, we observed an improvement of the MMCT efficiency 5.5 times for HT1080 cells (Supplementary Table S7). Next, we combined all modifications (Figure 1d) and compared a new MMCT protocol with the original procedure in HT1080 (Figure 3d). The HAC stability was verified by FISH (Figures 4g,h). This experiment was repeated three times in each of the following three cell lines: HT1080, HeLa, and mES (Table 3). In two cell lines, HT1080 and HeLa, the new MMCT protocol was six times more efficient than the original one (Table 3) (*F*(1,6) = 721.5, *P* < 0.0001). In mouse embryonic stem cells (mES), no HAC transfer events were detected using the original MMCT protocol. In contrast, seven HAC transfer events, averaging 2.3 colonies per experiment repeat, were detected when the new MMCT protocol was used (Table 3), and in this sense, the new MMCT protocol is a dramatic improvement.

### Cell cycle synchronization by a thymidine block does not improve the MMCT procedure

Despite using better drugs, the response of CHO cells to microtubule inhibitors was less that we had hoped. We wondered if CHO cells would respond better to an inhibitor that blocked a different part of the cell cycle. We selected the S phase inhibitor, thymidine, as it is relatively none toxic and its inhibitory action is rapidly reversible. Hence, our strategy to further improve the protocol was to first synchronizing the entire CHO population at S phase with a thymidine block, followed by release, a suitable period of incubation and finally induction of mitotic arrest using TN-16 and Griseofulvin as the majority of cells entered M phase, increasing the percentage of metaphase cells and consequently microcells production. Our results indicated that metaphase formation was increased slightly (1.5-fold) (Supplementary Table S8), but no improvement was achieved in the MMCT protocol (Supplementary Table S9).

### Comparison of HAC integrity using the modified and the original MMCT protocols

Cytochalasin B has been described to induce DNA fragmentation^48^ and at the concentrations of Cytochalasin B (20 µmol/l) used in MMCT^34,40,49^ could potentially induce damage to the HAC. Hence, we decided to examine if there is a difference in the level of HAC damage when the original MMCT protocol with Cytochalasin B versus the new protocol with Latrunculin B was applied. We performed Southern blot analysis of the structure of the alphoid array within the alphoid^tetO^-HAC after *Spe*I digestion^43^ (see Materials and Methods for details). A total of 36 HAC-containing clones, obtained from the original (18 clones) and new protocol (18 clones), were analyzed (Supplementary Figure S3a–c). The appearance of additional bands or bands disappearance in some clones on the Southern blot is in agreement with previous observations that recombination/rearrangements may occur within the HAC during the MMCT transfer. Though comparison of *Spe*I profiles revealed some advantage of the new protocol versus the original one (see Supplementary Figure S3d), we may conclude only that the modified MMCT protocol at least is not more damaging. Therefore, more investigative works needs to be done to evaluate how damaging the original versus new MMCT process is to the HAC to make a final conclusion.

All steps of the modified MMCT protocol are described in details in Supplementary Materials.

## Discussion

Since its development, MMCT remains the main method to selectively transfer natural and artificial chromosomes between differ vertebrate cell lines. The alternative methods that are not so commonly applied are based on physical purification of metaphase chromosomes.^50,51^ This is unfortunate as the low efficiency of chromosome transfer by MMCT impedes the wider use of MACs and HACs. This is particularly important as MACs are the logical choice for the next-generation vectors to be used in synthetic biology. MACs/HACs have an unlimited gene-carrying capacity.

The main work on optimizing the MMCT protocol has thus far been carried out by Mitsuo Oshimura’s lab at Tottori University. This group has mainly focused on the last step of MMCT, *i.e.*, the fusion of microcells with the recipient cells. In one work, to increase the chromosome transfer efficiency, the authors proposed the lipid envelope of an inactivated hemagglutinating virus of Japan (that has a similar effect on membrane fusion as polyethylene glycol) for microcell–cell fusion.^52^ The HN protein of the hemagglutinating virus of Japan recognizes acetyl type sialic acid as a receptor. Another modification involves the expression of H and F measles virus envelope proteins in the donor cells (*i.e.*, CHO).^53^ The H (hemagglutinin) and F (fusion) proteins mediate both viral attachment and membrane fusion. They bind to human CD46 surface receptor. The HAC transfer efficiency of this approach in HT1080 is comparable with the standard MMCT protocol using inactivated hemagglutinating virus of Japan viral envelope. This modified protocol is limited to recipient cell lines that express the CD46 receptor. A more recent work overcomes this limitation by fusing a targeting protein such as single-chain antibodies (scFv), peptides, growth factors, or cytokines to the extracellular C terminus of the H protein.^49^ So in principle, any cell type may be targeted as a recipient cell for MMCT. Oshimura’s group has also established a cryopreservation method to store microcells at −80 °C.^54^ This innovation significantly aids the MMCT protocol as preparation of microcells is time-consuming and not easily scaled up.

In our study, we also attempted to improve the efficiency of the MMCT transfer technology. However, our focus has been on the sections of the MMCT protocol that has thus far been neglected. We demonstrated that the replacement of key chemicals, Colcemid and Cytochalasim B for TN-16 + Griseofulvin and Latrunculin B in combination with a surface coating (Collage/Laminin), significantly increased the efficiency of HAC transfer to recipient cells. The modified MMCT protocol was successfully applied to four recipient cell lines including human fibrocarcoma HT1080, cervical cancer HeLa, human mesenchymal stem cells (hiMSC), and mouse embryonic stem (mES) cells. Southern blot analysis suggests that the modified MMCT protocol is less damaging compared with the standard protocol. However, to make a definitive conclusion, more HAC-containing clones have to be characterized.

The maximum increase in MMCT efficiency that we have observed in our experiments was about sixfold. It is obvious that further work to optimization of the MMCT protocols is required. The next step in further developing the MMCT protocol may lie in the improvement of purification of microcells and the combination of our protocol for microcell generation with the improved fusion and storage protocols developed by Oshimura’s group. To conclude, the improved MMCT technique for gene transfer via HAC vectors will facilitate the functional studies of genes and cell engineering such as reprograming of cells.

## Materials and Methods

### Cell culture

All cell culture media and components were purchased from Life Technologies, (Gaithersburg, MD) and Sigma (St. Louis, MO), unless other indicated. CHO cells were routinely maintained in 5% CO_2_ atmosphere in F12 medium supplemented with 10% fetal bovine serum, 100 U/ml penicillin, 100 mg/ml streptomycin, and 2 mmol/l l-glutamine. HT1080, HeLa, and hiMSC cell lines were routinely maintained in 5% CO_2_ atmosphere in Dulbecco’s modified Eagle’s medium (DMEM) supplemented with 10% fetal bovine serum, 100 U/ml penicillin, 100 mg/ml streptomycin, and 2 mmol/l l-glutamine. Murine ES cells (E14 Tg2a, BayGenomics) were cultured on gelatin-coated dishes in Knockout-DMEM supplemented with 15% ES cell-qualified fetal bovine serum, 100 U/ml penicillin, 100 mg/ml streptomycin, 2 mmol/l l-glutamine, nonessential amino acids, 50 μmol/l β-mercaptoethanol, and 1,000 U/ml leukemia inhibitory factor (LIF). For routine passaging, cells were washed in phosphate-buffered saline (PBS), treated with 0.25% Tripsine solution, and split at 1:4 ratio. 

### Preparation of metaphase spreads

Metaphase spreads were prepared as described previously^40^ with minor modifications. Exponentially growing (80% confluent) HAC-carrying cells were treated for 2–4 hours or overnight at 37 °C with 0.1 μg/ml Colcemid (KaryoMAX, Life Technologies) in 5% CO_2_ atmosphere. Cells were harvested by trypsinization and incubated in hypotonic (0.56% KCl) solution for 20 minutes. After that, cells were fixed in a fixative solution (methanol/acetic acid 3:1, v/v), washed three times in the fixative solution, and stored, if necessary, in fixative solution at −20 °C. For metaphase spreads, cells suspension was placed dropwise on precleaned fat-free microscope glass slides (Superfrost; Thermo Scientific, Darmstadt, Germany), air-dried, and aged at least overnight or longer at room temperature (RT) in dust-free place. 

### FISH with PNA probe

Slides with metaphases were rehydrated with 1× PBS for 15 minutes at RT, fixed with 4% paraformaldehyde, prepared on 1× PBS for 2 minutes, and washed three times with 1× PBS for 5 minutes. Then, slides were gradually dehydrated at RT for 5 minutes each: 70% ethanol (EtOH), 90% EtOH, 100% EtOH and dried up. Hybridization mix (10 mmol/l Tris–HCl, pH 7.4; 70% formamide; 5% dextran sulfate; 10 ng PNA–FITC–tetO; 10 ng PNA–TRITS–telomere) was applied onto each dehydrated slide in 20 μl of volume and covered with cover glass. After, slides were denaturated at 80 °C on heating table for 3 minutes (covered from light). Slides were incubated in darkness 2–6 hours at RT. After indicated period of time, cover glasses were removed, and slides were washed two times in Washing Solution I (70% formamide/10 mmol/l Tris–HCl, pH 7.4/0.1% bovine serum albumin) for 15 minutes, three times in Washing Solution II (20 mmol/l Tris–HCl, pH 7.4; 136 mmol/l NaCl; 0.08% Tween) for 5 minutes, and briefly rinsed once in PBS. Then, slides were gradually dehydrated at RT for 5 minutes each: 70% EtOH, 90% EtOH, 100% EtOH and dried up. Slides were mounted in Vectashield mounting media, containing 4′,6-diamidino-2-phenylindole (Vector Laboratories, Burlingame, CA). Images were captured and analyzed using a DeltaVision microscopy imaging system and software in the CRC, LRBGE Fluorescence Imaging Facility (NIH). The PNA probes used for FISH were provided by Panagen Company (Seoul, South Korea).

### Actin cytoskeleton disassembling test

About 5 × 104 CHO cells were plated onto gelatin-covered cover slips in 24-well plate and left overnight. At the next day, cells were washed with DMEM and treated with DMEM-diluted inhibitors of actin cytoskeleton formation; Cytochalasin D, Chaetoglobosin A, and Latrunculin B were diluted in concentrations 0.2, 2, and 20 μmol/l; Swinholide A was diluted in concentrations 0.1, 1, and 10 μmol/l; and Wiskostatin was diluted in concentrations 5, 50, and 500 μmol/l (also see Supplementary Figure S1). Cells were treated with the drugs for 30 minutes, then washed once with 1× PBS, and fixed with 4% paraformaldehyde on PBS 15 minutes at RT. After fixation, cells were stained with Rhadomine–Phalloidin solution prepared from Phalloidin–Tetramethylrhodamine Conjugate stock solution 1,000× (Santa Cruz, Dallas, TX). Cells were washed once with PBS and stained with 250 μl of stained solution prepared on PBS (5 μl of stock solution to 5 ml of PBS) for 30 minutes at RT in the dark. At the end of indicated time, cells were washed twice with 1× PBS, and cover slips with fixed stained cells were mounted on microscopic slides upside down in mounting media (36,935 molecular probes). Slides were analyzed were using a DeltaVision microscopy imaging system and software in the CRC, LRBGE Fluorescence Imaging Facility (NIH).

### Colony staining and counting

After MMCT, colony of cells were stained with crystal violet for counting. Dishes with cells were washed twice with PBS. Cells were fixed with 4% paraformaldehyde on PBS 15 minutes at RT. After fixation, cells were stained with crystal violet (prepared in 10% ethanol) for 15 minutes at RT. After staining, dishes were gently washed with deionized water until the water no longer runs blue. Images of stained colonies were captured and analyzed using Gel-Doc Documentation System and Quantity One Software (Bio-Rad, Hercules, CA).

### Drug analysis for metaphase preparation in hamster CHO cells

About 10 µg/ml Colcemid was purchased from Thermo Fisher Scientific. All other microtubule inhibitors were purchased from Santa Cruz Biotechnology. The following stock solutions of microtubule inhibitors were prepared and kept at −20 °C; TN-16 80 mmol/l in dimethyl sulfoxide (DMSO), Griseofulvin 25 mmol/l in DMSO, Nocodazole 25 mmol/l in DMSO, Vinorelbine ditartarate 25 mmol/l in DMSO, Noscapine hydrochloride 25 mmol/l in water, and Myoseverin B 2.5 mmol/l in DMSO. CHO cells were plated onto 100-mm treated cell culture dishes (Denville, Holliston, MA) at 40% confluence and allowed to grow to 70% confluence. The cell cultures were then washed once with PBS and 10 ml of fully supplemented F12 culture medium and the appropriate concentration of microtubule inhibitors added. The plate was then left to incubate for 18 hours. Next, the spent medium was removed and the number of floating cells within was counted using a hemocytometer. The plate was then washed with 10 ml of PBS, and adherent cell was removed by incubation with 1 ml of trypsin. Nine milliliter of F12 media was added, and the cells were washed off. Cell count of adhere cell was made using a cell counter (Auto T4, Nexcelom) and resuspended in 50 mmol/l of KCl solution for 20 minutes at 37 °C. After that, cells were fixed by three washes of fixative solution (methanol/acetic acid 3:1, v/v). Fixed cells were dropped onto microscope slides and mounted in Vectashield mounting media, containing 4′,6-diamidino-2-phenylindole (Vector Laboratories). Images of cells were obtained using a DeltaVision microscopy imaging system at the CRC, LRBGE Fluorescence Imaging Facility (NIH), and the percentage of metaphase in total cell population was counted using ImageJ.

### Cultural plastic modifications for metaphase preparation in hamster CHO cells

About 3,090 µg/ml Collagen I, bovine (sc-29009), 1,960 µg/ml Laminin (sc-29012), and 500 µg/ml Fibronectin (sc-29011) were purchased from Santa Cruz Biotechnology. About 0.01% solution of Polylysine (P4707) was purchased from Sigma–Aldrich. To each 60-mm dish (Sarstedt, Nümbrecht, Germany), cell adhesion factors diluted in the appropriate buffers were added; 51 µl of 3,090 µg/ml Collagen in 2,950 µl of 20 mmol/l acetic acid; 39 µl of 1,960 µg/ml Laminin in 2,960 µl of PBS, pH 7.4; 300 µl of 500 µg/ml Fibronectin in 2,700 µl of PBS, pH 7.4; 1,500 µl of 0.01% Polylysine in 1,500 µl of PBS, pH 7.4. The plates were left overnight at RT and later washed three times with 3 ml of PBS. CHO cells were then added. Once 70% cell confluence was achieved the culture was blocked at metaphase by overnight incubation with 50 µmol/l Griseofulvin and 160 µmol/l TN-16. The spent media were then removed, and the plate was washed once with PBS. The culture was then trypsinized, resuspended in 50 mmol/l of KCl solution for 20 minutes at 37 °C, fixed by three washes of fixative solution (methanol/acetic acid 3:1, v/v), spotted onto microscope slides, and mounted in Vectashield mounting media, containing 4′,6-diamidino-2-phenylindole (Vector Laboratories). Slides were analyzed using a DeltaVision microscopy imaging system (LRBGE Fluorescence Imaging Facility, NIH), and the percentage of metaphase in total cell population was counted using ImageJ.

### Southern blot hybridization analysis

Southern-blot hybridization was performed with a ^32^P-labeled probe as described previously^43^ with minor changes. Genomic DNA was prepared in agarose plugs and restriction-digested either by *Spe*I in the buffer recommended by the manufacturer. The digested DNA was CHEF (CHEF Mapper, Bio-Rad) separated (autoprogram, 10–70 kb range, 22-hour transfer), transferred to membrane (Amersham Hybond-N+), and blot-hybridized with a 201-bp probe specific for the YAC/BAC vector sequence in the alphoid^tetO^-HAC. This sequence is present in multiple copies in the HAC. DNA sequence for the probe was amplified by PCR using the primers indicated here (FWD: 5′-GGGCAATTTGTCACAGGG-3′; REV: 5′-ATCCACTTATCCACGGGGAT-3′). Blot was incubated for 2 hours at 65 °C in prehybridization Church’s buffer (0.5 mol/l Na-phosphate buffer containing 7% sodium dodecyl sulfate (SDS) and 100 µg/ml of unlabeled salmon sperm carrier DNA). The labeled probe was heat denatured in boiling water for 5 minutes and snap cooled on ice. The probe was added to the hybridization buffer and allowed to hybridize overnight at 65 °C. Blot was washed twice in 2× saline sodium citrate buffer (SSC) (300 mmol/l NaCl, 30 mmol/l sodium citrate, pH 7.0), 0.05% SDS for 10 minutes at RT; then twice in 2× SSC, 0.05% SDS for 5 minutes at 60 °C; twice in 0.5× SSC, 0.05% SDS for 5 minutes at 60 °C; and twice in 0.25× SSC, 0.05% SDS for 5 minutes at 60 °C. Blot was exposed to X-ray film for 24–72 hours at −80 °C.

## Figures and Tables

**Table 1:** MMCT efficiency: Colcemid versus TN-16 + Griseofulvin combination

		*Microtubule inhibitors*						
		*Colcemid (*N* of colonies)*			*TN-16 + Griseofulvin^a^ (*N* of colonies*)			*Improvement*
*Number*	*Cell line*	*Exp1*	*Exp2*	*Exp3*	*Exp1*	*Exp2*	*Exp3*	*Mean ± SD*
1	HT1080	49	42	45	188	155	174	3.80 ± 0.09
2	HeLa	34	67	52	122	258	195	3.73 ± 0.13
3	hiMSC	2	4	4	7	15	12	3.42 ± 0.38

Replacement of Colcemid with TN-16 + Griseofulvin significantly increased MMCT efficiency (ANOVA *F*(1,6) = 80.35, *P* = 0.0001). ANOVA, analysis of variance; MMCT, microcell-mediated chromosome transfer.

a Number of colonies.

**Table 2:** MMCT efficiency: different actin cytoskeleton disassembling drugs

		*Actin inhibitors*												
		*Cytochalasin B (*N* of colonies)*			*Cytochalasin D (*N* of colonies)*			*Chaetoglobosin A (*N* of colonies)*			*Latrunculin B (*N* of colonies)*			*Improvement*
*Number*	*Cell line*	*Exp1*	*Exp2*	*Exp3*	*Exp1*	*Exp2*	*Exp3*	*Exp1*	*Exp2*	*Exp3*	*Exp1*	*Exp2*	*Exp3*	*Mean ± SD*
1	HT1080	50	52	55	98	100	94							1.86 ± 0.14^a^
2	HT1080	49	57	58				65	68	69				1.24 ± 0.08
3	HT1080	40	54	47							118	154	156	3.04 ± 0.25^b^

ANOVA was used (*F*(2, 6) = 12.83). MMCT efficiency significantly increased when Cytochalasin B was replaced with either Cytochalasin D^a^ (*P* = 0.0006) or Latrunculin^b^ (*P* < 0.0001). No significant increase was detected when Cytochalasin D was used (*P* = 0.1879). ANOVA, analysis of variance; MMCT, microcell-mediated chromosome transfer.

**Table 3:** MMCT efficiency: an original versus a new protocol

		*Protocol*						
		*Original (*N* of colonies)*			*New (*N* of colonies)^a^*			*Improvement*
*Number*	*Cell line*	*Exp1*	*Exp2*	*Exp3*	*Exp1*	*Exp2*	*Exp3*	*Mean ± SD*
1	HT1080	57	60	56	345	355	330	5.95 ± 0.09
2	HeLa	35	40	48	213	232	283	5.93 ± 0.15
3	mES	0	0	0	2	2	3	n/c

The new MMCT protocol is significantly more efficient than the original (ANOVA *F*(1,6) = 721.5, *P* < 0.0001). ANOVA, analysis of variance; MMCT, microcell-mediated chromosome transfer; n/c, not countable.

## Notes

The first two authors contributed equally to this work.
