# Supplementary material for: Moving toward a higher efficiency of microcell-mediated chromosome transfer
Source: Mol Ther Methods Clin Dev. 2016 Jun 22;3:16043–. doi: 10.1038/mtm.2016.43 (PMC4916947; doi:10.1038/mtm.2016.43)
Supplement: Supplementary Tables and Figures [file mtm201643-s1.pdf]

Supplementary Table S1: Metaphase induction by a single tubulin inhibitor

Drug concentration	% Adherent cells	#Metaphase/ #Total cells	% Metaphase	Ratio*
269nM Colcemid (Control – 100ng/ml)	82	30 / 805	**3.7**	1.1
269nM Colcemid (Control – 100ng/ml)	72	16 / 517	**3.1**	0.9
80µM TN16	92	4 / 655	0.6	0.2
160µM TN16	81	18 / 551	3.3	1.0
25µM Nocodazole	85	38 / 1650	2.3	0.7
50µM Nocodazole	87	12 / 862	1.4	0.4
25µM Vinorelbine ditartrate	96	10 / 1225	0.8	0.2
50µM Vinorelbine ditartrate	85	3 / 1433	0.2	0.1
25µM Griseofulvin	94	26 / 787	3.3	1.0
50µM Griseofulvin	93	47 /833	**5.6**	1.6
100µM Griseofulvin	90	43 / 925	4.6	1.4
200µM Griseofulvin	89	41 / 711	5.8	1.7

Colcemide, TN16 and Nocodazole are microtubule destabilizers that bind to the same site on the α-β tubulin dimer, the colchicine site. Vinorelbine binds to the vinca site on the tubulin dimer. The tubulin binding site of griseofulvin is uncertain.

* The relative ratio between the percentage metaphase obtained from the sample drug to the average percentage metaphase from treatment with 100ng/ml colcemide

Supplementary Table S2: Metaphase induction by tubulin inhibitors from two classes

**Drug concentration**	**% Adherent cells**	**#Metaphase/ #Total cells**	**% Metaphase**	**Ratio***
50µM Griseofulvin + 160µM TN16	85	124 / 827	15.0	**4.4**
50µM Griseofulvin + 25µM Nocodazole	57	200 / 1256	15.9	**4.7**
50µM Griseofulvin + 100µl Colcemid	72	56 / 643	8.7	2.6
50µM Griseofulvin + 1.6nM Vinorelbine ditartrate	95	13 / 477	2.7	0.8
50µM Griseofulvin + 8nM Vinorelbine ditartrate	93	8 / 346	2.3	0.7
50µM Griseofulvin + 40nM Vinorelbine ditartrate	90	33 / 729	4.5	1.3
50µM Griseofulvin + 200nM Vinorelbine ditartrate	89	9 / 509	1.8	0.5
50µM Griseofulvin + 1µM Vinorelbine ditartrate	87	14 / 741	1.9	0.6
50µM Griseofulvin + 5µM Vinorelbine ditartrate	89	19 / 1012	1.9	0.6
50µM Griseofulvin + 50µM Vinorelbine ditartrate	91	8 / 502	1.6	0.5
50µM Griseofulvin + 100µM Vinorelbine ditartrate	50	0 /150	0.0	0.0

* The relative ratio between the percentage metaphase obtained from the sample drug to the average percentage metaphase from treatment with 100ng/ml Colcemide

Supplementary Table S3: Metaphase induction by Griseofulvin and by increasing a concentration of TN16

Drug concentration	% Adherent cells	#Metaphase/ #Total cell	% Metaphase	Ratio*
50µM Griseofulvin + 160µM TN16	90	182 / 1765	10.3	1.0
50µM Griseofulvin + 240µM TN16	87	112 / 1134	9.9	1.0
50µM Griseofulvin + 320µM TN16	85	177 / 1444	12.3	1.2

* The relative ratio between the percentage metaphase obtained from the standard concentration of TN16 (160µM) used and increasing concentrations of TN16.

Supplementary Table S4: Metaphase induction with three classes of tubulin inhibitors

Drug concentration	#Metaphase/ #Total cell	% Metaphase	Ratio*
50µM Griseofulvin + 160µM TN16	352 / 2081	16.9	1.0
50µM Griseofulvin+60µM TN16+12.5µM Noscapine HCl	161 / 1091	14.8	0.9
50µM Griseofulvin+160µM TN16+25µM Noscapine HCl	215 / 1594	13.5	0.8
50µM Griseofulvin+160µM TN16 + 100µM Noscapine HCl	161 / 1178	13.7	0.8
50µM Griseofulvin+160µM TN16+5µM Myoseverin B	237 / 1832	12.9	0.8
50µM Griseofulvin+160µM TN16+2.5µM Myoseverin B	160 / 1040	15.4	0.9
50µM Griseofulvin+160µM TN16+0.5µM Myoseverin B	117 / 810	14.4	0.9
50µM Griseofulvin+160µM TN16+0.1µM Myoseverin B	88 / 830	10.6	0.6
50µM Griseofulvin+160µM TN16+5pM Maytansine	127 / 943	13.5	0.8
50µM Griseofulvin+160µM TN16+25pM Maytansine	121 / 733	16.5	1.0
50µM Griseofulvin+160µM TN16+50pM Maytansine	237 / 1512	15.7	0.9
50µM Griseofulvin+160µM TN16+100pM Maytansine	238 / 1330	17.9	1.1

* The relative ratio between the percentage metaphase obtained from the standard drug combination (50µM Griseofulvin + 160µM TN16) and other drug combinations tested.

Supplementary Table S5: Metaphase induction by different surface coatings with 50µM Griseofulvin and 160µM TN16

Drug concentration	#Metaphase/ #Total cells	% Metaphase	Ratio*
Control plastic (Exp 1)	308 / 2830	10.9	1.0
Control plastic (Exp 2)	106 / 986	10.8	1.0
Collagen (Exp 1)	296 / 2524	11.7	1.1
Collagen (Exp 2)	33 / 286	11.5	1.1
Fibronectin (Exp 1)	36 /333	10.8	1.0
Fibronectin (Exp 2)	273 / 2966	9.2	0.8
Laminin (Exp 1)	241 / 2178	11.1	1.0
Laminin (Exp 2)	30 / 236	12.7	1.2
Polylysine (Exp 1)	130 / 1575	8.3	0.8
Polylysine (Exp 2)	51 / 680	7.5	0.7
Collagen + Fibronectin	159 / 1931	8.2	0.8
Collagen + Laminin	221 / 1568	14.1	**1.3**
Collagen + Polylysine	228 / 2183	10.4	1.0
Collagen + Fibronectin+ Polylysine (CFP)	178 / 1619	11.0	1.0
Collagen + Fibronectin + Laminin (CFL)	150 / 1811	8.3	0.8
Collagen + Polylysine+ Laminin (CPL)	173 / 1184	14.6	1.3

* The relative ratio of percentage metaphase between the standard plastic surface and surfaces coated with cell adhesion proteins.

Supplementary Table S6: MMCT efficiency: Plastic vs Collagen/Laminin surface coating

		**Plastic modification**						**Improvement**
		**No coating**			**Collagen/Laminin coating**			
#	Cell line	Exp1	Exp2	Exp3	Exp1	Exp2	Exp3	Mean ± S.D
1	HT1080	60	58	64	96	98	104	1.64 ± 0.05

Supplementary Table S7: MMCT efficiency: Original vs new drugs combinations

		**Chemical**						**Improvement**
		**Colcemid Cytochalasin B**			**TN16+Griseofulvin Latranculin B**			
#	Cell line	Exp1	Exp2	Exp3	Exp1	Exp2	Exp3	Mean ± S.D
1	HT1080	55	53	50	283	290	295	5.51 ± 0.38

Supplementary Table S8: Improvement of metaphase induction cell synchronization using a thymidine block

Drug concentration	#Metaphase/ #Total cells	% Metaphase	Ratio***
T+G* (No coating: Control)	192 /1847	10.4	1.0
T+G<br>21hr Thy + 0 hr recovery in F12	111 / 1264	8.8	0.8
T+G<br>21hr Thy + 4hr recovery in F12	135 /1185	11.4	1.1
T+G<br>21hr Thy + 6hr recover in F12	203 /1317	15.4	1.5
T+G<br>21hr Thy + 8hr recovery in F12	238 /1442	16.5	1.6
**Control**<br>C+L**, T+G	217 / 1472	14.7	1.0
**Exp1**<br>C+L, T+G<br>19hr Thy + 8.5hr recovery	261 /1236	21.1	1.4
**Exp2**<br>C+L, T+G<br>19hr Thy + 8.5hr recovery	232 / 1001	23.2	1.6
**Exp3**<br>C+L, T+G<br>19hr Thy + 8.5hr recovery	179 / 920	19.3	1.3

* T+G is 160μM TN16 and 50μM Griseofulvin.
** C+L is Collagen and Laminin coating on the plastic surface of the tissue culture plate.
*** The relative ratio of percentage metaphase between controls and synchronized cultures.

Supplementary Table S9: MMCT efficiency: Use of thymidine block and cell synchronization.

		Combined modifications						
		**Colcemid Cytochalasin B**			**TN16+Griseofulvin Latranculin B Collagen/Laminin Thymidine Block**			**Improvement**
#	Cell line	Exp1	Exp2	Exp3	Exp1	Exp2	Exp3	Mean ± S.D
1	HT1080	55	52	57	328	323	316	5.91 ± 0.34

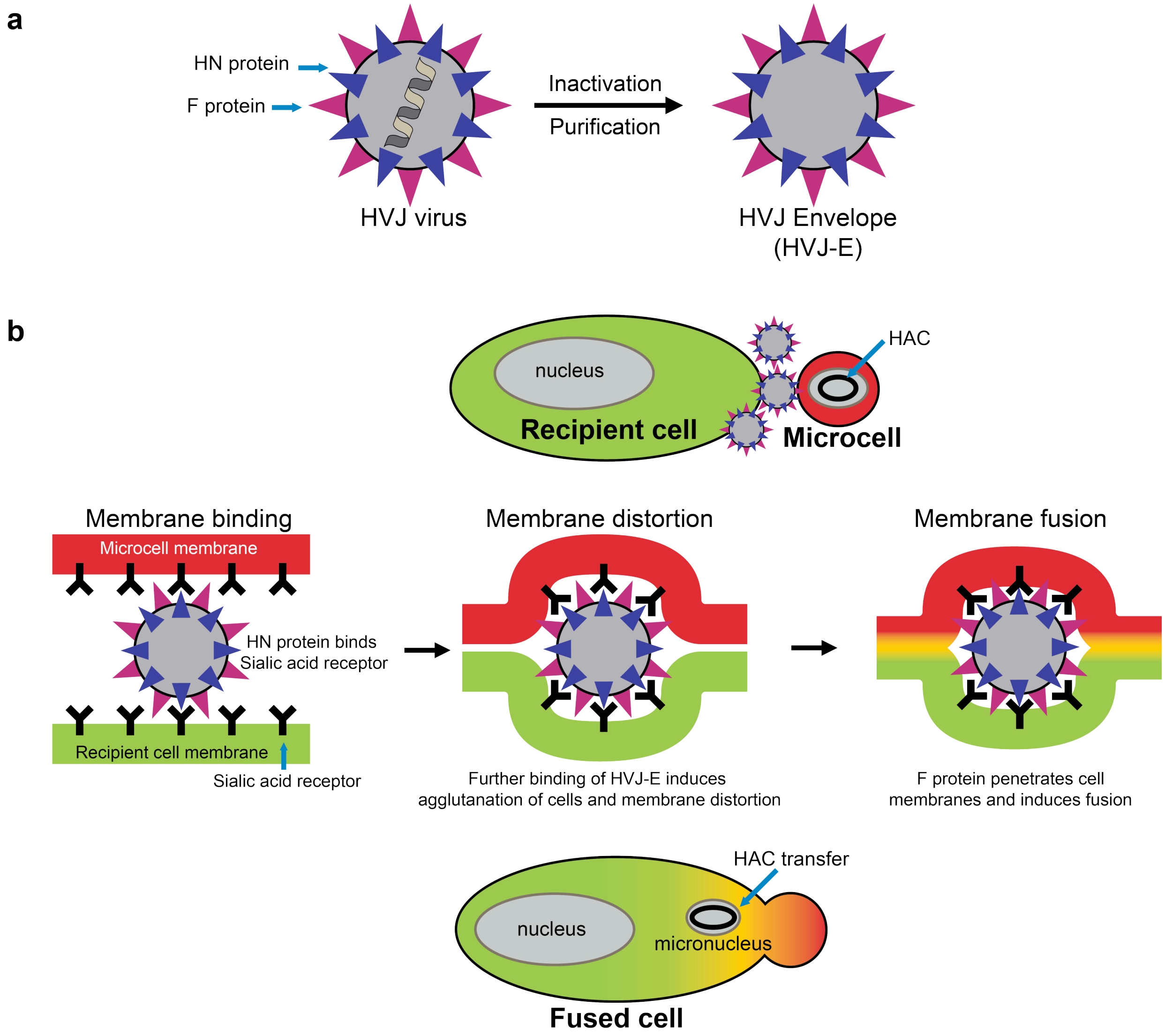

Supplementary Figure S1: Scheme of microcell-recipient cell fusion induced by inactivated Hemagglutinating Virus of Japan (HVJ)-envelope. (**a**) HVJ Envelope (Cat: ISK-GN-001-EX, CosmoBio) is a viral vesicle obtained from complete inactivation of Sendai virus (HVJ) that still retains cell membrane-fusing capability. Two HVJ proteins are of importance, the Hemagglutinin-neuraminidase (HN) protein which binds to the sialic acid receptor and Fusion (F) protein which induces cell membrane fusion. (**b**) Fusion of a microcell to a recipient cell is induced by the incubation with the HVJ-envelope. In the membrane binding step, the HVJ-E vesicles bind to the membrane of both a microcell and a recipient cell via the viral HN protein which recognizes the sialic acid receptor. The Membrane distortion step follows as the cells undergo agglutination cross-linking due to the HVJ-E particle. Membrane fusion begins once the hydrophobic domain at the N-terminal of the F protein (F1) penetrates the double lipid layer of the cell membrane and induces a transient but severe alteration of the cell membrane structure which is aided by heating the cell/HVJ-E complex at 37°C (Based on product information from CosmoBio). Fusion of the microcell to the recipient cell, releases the micronuclei into the cytoplasm. It is believed that the micronuclei will reintegrate into the host nucleus at the next cell cycle.

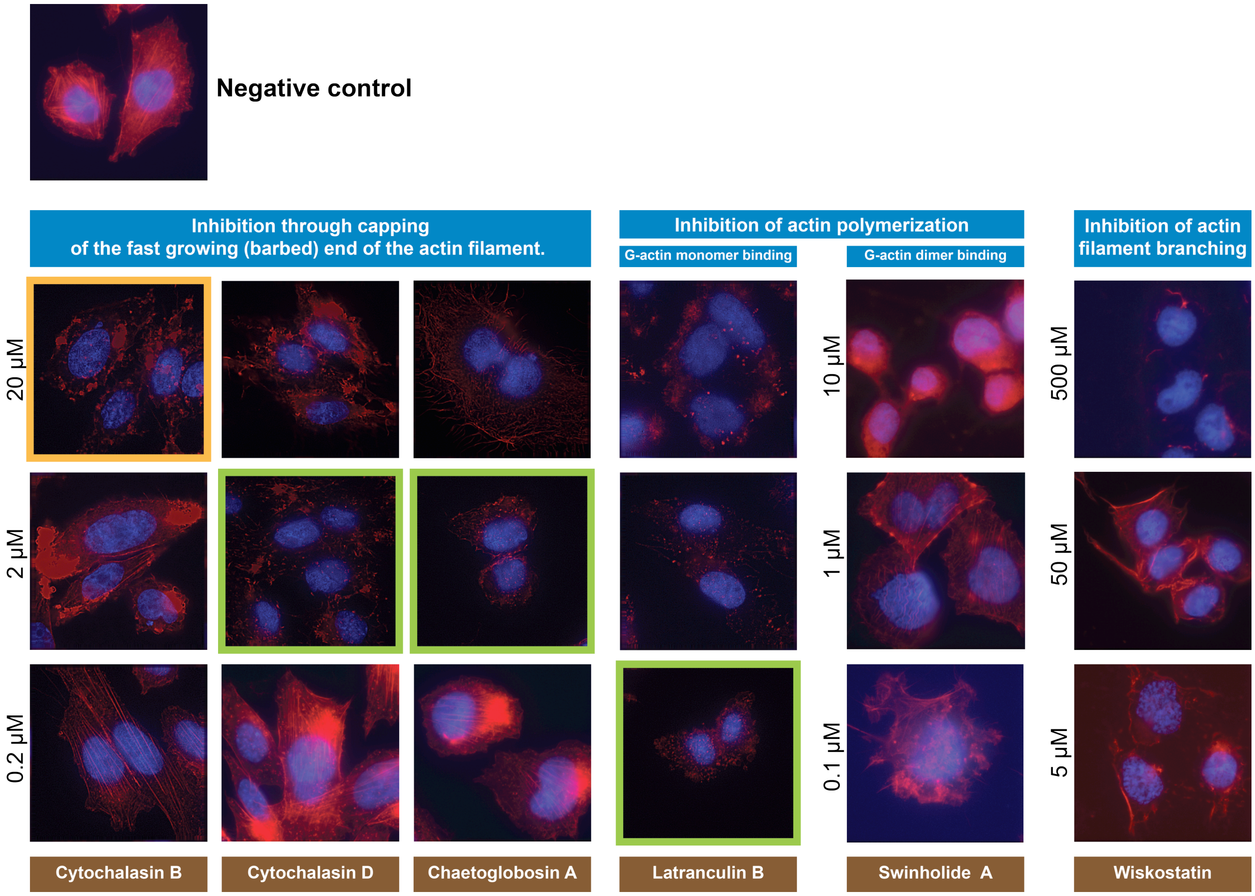

Supplementary Figure S2: Actin cytoskeleton disarrangement in CHO cells induced by actin inhibitors used in this study at different concentrations. The actin inhibitors we used can be grouped into three families by mode of action; Capping of the actin filament; Inhibition of actin monomer polymerization and inhibition of actin filament branching. For detailed procedure of this experiment – see Materials and Methods. The yellow frame indicates the drug and concentration used in the original MMCT protocol (20µM Cytochalasin B). Green frames indicate potential drug candidates to replace Cytochalasin B.

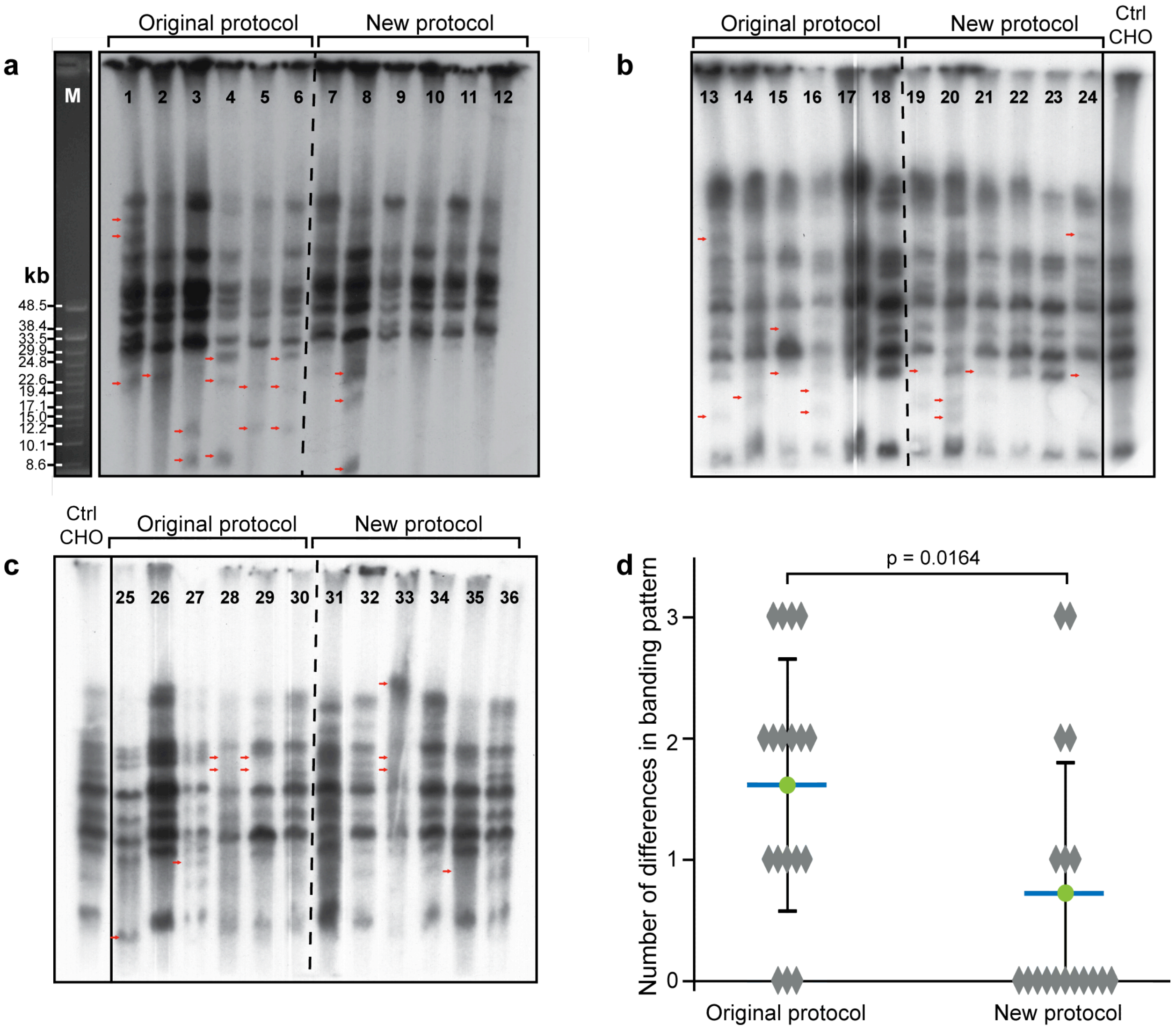

Supplementary Figure S3: Southern-blot analysis of the alphoid^{tetO}-HAC in clones obtained by the original and modified MMCT protocols. Wells (**a**) 1-6, (**b**) 13-18 and (**c**) 25-30 correspond to the clones derived from MMCT using Colcemid and Cytochalasin B as key components of the MMCT procedure. Wells (**a**) 7-12, (**b**) 19-24 and (**c**) 31-36 correspond to clones derived from MMCT using Griseofulvin plus Latrunculin B as key components of the MMCT procedure. The control (Ctrl-CHO) is the HAC containing CHO host cell line that has not undergone the MMCT procedure. The membrane was probed with the YAC/BAC vector sequence used for assembly of the alphoid^{tetO}-HAC. This sequence was amplified more than 30 times during HAC formation. Different copies of this sequence within a 1.1 Mb alphoid DNA array are visualized by hybridization after digestion of genomic DNA by SpeI. (**d**) A decrease in HAC rearrangements was observed in clones derived from the new protocol compared to the original protocol, Unpair t-test [t(34) = 2.5261 p = 0.0164].

A modified MMCT chromosome transfer protocol

Step 1a Preparation for MMCT, 2 days

1) Make medium for donor CHO cells: F12, 10% FBS, P/S, Glutamine, BSD 10 μg/ml.
2) Make medium for the desired recipient cell line.
3) Cover cultural flasks (Nunc T24) with collagen/laminin.
4) Incubate the flasks with collagen solution (50 μg/ml in 20 mM acetic acid) overnight at RT.
5) Remove collagen solution.
6) Gently wash the flasks with 1x PBS 3 times.
7) Add laminin solution (25 μg/ml in PBS) and incubate overnight at RT.
8) Wash the flasks with 1x PBS 3 times.
9) Assemble 4 of 8 μm (Millipore, TETP02500), 4 of 5 μm (Millipore, TMTP02500) and 4 of 3 μm (Millipore, TSTP02500) filters in Swinnex filter holders (Millipore, SX0002500).
10) After that, wrap them with aluminum foil, autoclave for 40 min and then dry for 2-3 hrs.

Step 1b Recipient and donor cells preparation, 4 days

11) Grow up the desired numbers of recipient cells (3-5 $x10^6$). Cells should be collected at 80% of confluence from culture dish.
12) Grow up to 12 flasks of donor cells (80% confluent) with 3 ml medium per a flask.
13) When the CHO cells grow up to 12 flasks, change the culture medium to medium containing cytostatic cocktail: 160 mM TN-16 (SantaCruz, sc-204347) and 50 mM Griseofulvin (SantaCruz, sc-202171).
14) Treat the CHO donor cells with cytostatic cocktail for 72 hrs. Change culture medium every day with a fresh portion of cytostatic cocktail.

Step 2 Microcell preparation, 1 day

15) Prepare Latranculin B medium by adding 40 μl of 2 mM Latranculin B (Santa Cruz, sc-203318) into 400 ml of DMEM.
16) Warm the Latranculin B-containing medium up to 37°C.
17) Collect cell culture media from all flasks with donor cells, centrifuge at 1200 rpm for 3 min and put the cells back into Latranculin B-containing medium to the flasks.
18) Fill each flask up to neck with Latranculin B-containing medium (60-61 ml of medium per a flask).
19) Closely wrap each neck and cap of the flask with parafilm 3 times to protect from water contamination.
20) Place each T24 flask into a 0.5 L spin bottle with a wide neck and fix them with a glue-paper at the fat side of the flask towards to the rotor position, and fill each of the spin bottles with the fixed flask with pre-warmed (37°C) distilled H_2O (130 ml per each bottle). Each bottle should weigh approximately 280 g. Check that they are balanced.
21) Spin water filled bottles, containing the flasks, for 60 min at 34°C at 8000 rpm.
22) Microcell pellets become visible in the angles of the flask. After spinning, take off all Latranculin B-containing medium.

NOTE: Don't aspirate the microcell pellet off!!!

23) Add 1 ml of warm serum free DMEM medium to each flask and re-suspend the microcell pellet completely.
24) Transfer the re-suspended microcells into a 50 ml falcon tube.
25) Wash the flasks once again with 1 ml of DMEM and collect the microcells in the same tube.
26) Spin microcells suspension at 3000 rpm for 5 min.
27) Re-suspend the microcell pellet in 1 ml of poured supernatant using 1 ml of cell culture sampler.
28) Adjust volume until 48 ml with serum-free DMEM.
29) Divide the common microcell suspension to 4 equal portions.
30) Filter each of the microcell portions sequentially through prepared 8µm→5µm→3µm filters from point 28.
31) After filtration, centrifuge the tubes at 3000 rpm for 5 min.
32) After centrifugation, re-suspend each pellet completely in 1 ml of serum-free DMEM.
33) Combine them all together and adjust a volume till 10 ml and then re-suspend carefully and centrifuge again at 3000 rpm for 5 min.
34) After centrifugation, place the tube with collected microcells under the hood.

NOTE: Now clean microcells are ready for fusion. A microcell pellet should be visible.

35) Aspirate medium from the recipient cells.
36) Wash once with PBS and harvest the cells by trypsin treatment.
37) Count the cells and take 3-5 x 10^6 cells for 2 vials (one – for fusion, another – for a negative control).
38) Centrifuge the cells at 1200 rpm for 3 min.
39) Wash the cells 2 times with serum-free DMEM (re-suspend the cells completely in 10 ml of DMEM each time and spin them down).

NOTE: Now clean recipient cells are ready for fusion.

Step 3 Fusion procedure with "HVJ Envelope Cell Fusion Kit" (Cosmo Bio ltd, Japan)

40) Dilute the cell fusion buffer (20x concentrate; stored at +4°C) 1:20 with a sterile pure ice cold water (e.g., endotoxin-free water for injection or ddH_2O from Gibco).
41) Prepare 2 ml of 1x cell fusion buffer on ice.
42) Suspend the microcells (from point 34) and the recipient cells (from point 39) pellets separately in 0.5 ml of ice-cooled cell fusion buffer (one-fold concentrate) using 2 ml tubes (preferably round-bottom tubes).
43) Centrifuge both pellets at 3000 rpm for 3 min at +4°C using centrifuge in a cold-room or pre-cooled centrifuge.
44) Suspend the recipient cells and the microcells pellets separately in ice-cooled cell fusion buffer (one-fold concentrate) at a concentration of 2 x 10^5 cells/25 µl.
45) Re-suspend 3 x10^6 recipient cells in 375 µl of cell fusion buffer and the microcell pellet in the same volume.

46) Mix both suspensions together by pipetting.
47) Place one 10 µl aliquot of HVJ-E suspension from -80°C on ice and add ice-cooled HVJ-E suspension to the mixed cells suspension (point 46) and mix by tapping.
48) Leave the mixture on ice for 5 min to allow HVJ-E to be adsorbed on the cell surface.
49) Incubate at 37°C for 15 min (mix by tapping every 5 min to induce cell fusion).
50) After 15 min, centrifuge a mixture at RT at 3000 rpm for 3 min to remove residual viral proteins.
51) Re-suspend the fused cells in 10 ml of the full incubating medium and place in the cultural dish.

Step 4 Colonies selection, 7-10 days

52) Start Blasticidin S (BSD) (Life Technologies, A11139-03) (5-10 µg/ml) selection after 24 hrs of incubation.
53) Observe BSD-resistant clones approximately after 1-1.5 week of selection.
